# The Impact of the COVID-19 Pandemic on Inpatient Admissions for Psychotic and Affective Disorders: The Experience of a Large Psychiatric Teaching Hospital in Romania

**DOI:** 10.3390/healthcare10081570

**Published:** 2022-08-18

**Authors:** Vlad Dionisie, Adela Magdalena Ciobanu, Emanuel Moisa, Mihnea Costin Manea, Maria Gabriela Puiu

**Affiliations:** 1 Department of Psychiatry and Psychology, “Carol Davila” University of Medicine and Pharmacy, 020021 Bucharest, Romania; 2 Department of Psychiatry, “Prof. Dr. Alexandru Obregia” Clinical Hospital of Psychiatry, 041914 Bucharest, Romania; 3 Discipline of Psychiatry, Neurosciences Department, “Carol Davila” University of Medicine and Pharmacy, 020021 Bucharest, Romania; 4 Department of Anaesthesia and Intensive Care Medicine, Faculty of Medicine, “Carol Davila” University of Medicine and Pharmacy, 020021 Bucharest, Romania

**Keywords:** COVID-19, pandemic, lockdown, inpatient, admission, psychiatry, involuntary, psychotic disorder, affective disorder

## Abstract

The COVID-19 pandemic resulted in a global sanitary crisis and, in addition, elicited serious mental health consequences. The utilization of psychiatric hospital-based services acts as an indicator of public mental health. Therefore, this research sought to investigate differences in the numbers and characteristics of inpatient admissions for psychotic and affective disorders at the largest Romanian psychiatric hospital between the period of lockdown (16 March–15 May 2020) and another three corresponding periods: the same year in the pre-lockdown period (16 January–15 March 2020), the immediate post-lockdown period (16 May–15 July 2020), and two years later (16 March–15 May 2022). A retrospective analysis was performed. The study included a total of 6604 patients. Inpatient admissions decreased during lockdown in comparison with the pre-lockdown period and immediate post-lockdown period for psychotic disorders (*p* < 0.001 and *p* < 0.001, respectively) and affective disorders (*p* < 0.001 and *p* < 0.001, respectively). For both psychotic and affective disorders, a decrease in the age of the patients admitted during lockdown, as compared with the pre-lockdown period (*p* < 0.05 and *p* < 0.001, respectively), was observed. The length of the hospital stay for affective disorders was higher immediately post-lockdown in comparison with the lockdown period (*p* < 0.001). Collectively, the present findings provide a glimpse of the immediate and long-term consequences of the COVID-19 pandemic and lockdown measures on patients’ access to mental healthcare in the form of hospitalization, and these findings could provide the basis for the development of a different approach to times of crisis.

## 1. Introduction

Beginning in December 2019, an emerging infectious disease, known as coronavirus infectious disease-2019 (COVID-19), started to rapidly spread worldwide, leading to an unprecedented global crisis. Soon after, in March 2020, the World Health Organization officially declared COVID-19 as a global pandemic [1]. As a result, numerous strategies, including physical distancing, travel restrictions, and even partial or complete lockdowns, were implemented by governments in order to limit the spread of the COVID-19 virus [2]. In Romania, the COVID-19 pandemic was officially declared on the 16 March 2020, when national authorities instituted a state of emergency and imposed lockdown measures [3]. Lockdown restrictions were gradually suspended from 15 May 2020 onwards. 

In addition to introducing serious changes into people’s daily lives, it was predicted from the early stages of the pandemic that the COVID-19 outbreak would have both acute and prolonged impacts on mental health [4,5]. Numerous studies demonstrated that the pandemic increased generic psychological distress, or even the prevalence of anxiety and depressive disorders, insomnia, post-traumatic stress disorder, and eating disorders [6,7,8,9]. Patients with COVID-19 represent a novel particular group of individuals who are at risk of developing psychiatric disorders, since they experience high levels of stress and use maladaptive coping strategies [10]. Moreover, there are serious concerns regarding the possibly raised risk of transient psychosis in healthcare workers [11]. In this scenario, an increase in psychiatric referrals to emergency services was expected. On the contrary, several studies observed a reduction in the number of psychiatric emergency department consultations and acute hospitalizations [12,13,14,15,16].

In addition to its direct effects, the outbreak has had indirect impacts on healthcare services for other diseases, including psychiatric services [17]. In Romania, similar to other countries, the mental health services underwent an important reorganization during lockdown. The standard in-person service in outpatient clinics was greatly curtailed or cancelled, and the majority of clinics shifted their services towards remote consultations, the day hospitals were closed, and the inpatient units’ capacities were reduced [14,17,18]. The activity of the “Prof. Dr. Alexandru Obregia” Clinical Hospital of Psychiatry in Bucharest was subjected to all of these measures. Throughout the pandemic period, there was no disruption in the provision of inpatient psychiatric care. Moreover, since the beginning of the pandemic, special wards have been established in the hospital for the isolation and treatment of patients with a dual diagnosis of a psychiatric disorder and COVID-19 (asymptomatic or mild/moderate symptoms).

Taking all of the above into consideration, there is an urgent need to explore the effects of the increased morbidity of mental health problems and the shortcomings in the provision of psychiatric services during the COVID-19 pandemic. Research regarding the utilization of inpatient psychiatric facilities can contribute to the overall picture of the effects of the COVID-19 pandemic and can provide important information regarding changes experienced by individuals with severe mental illnesses. Moreover, such data can aid in the development of much-needed future public policies aimed at addressing the changing needs of the population in terms of mental health [16,19].

In the present study, our aim was to evaluate the effects of the COVID-19 lockdown on inpatient admissions for major psychiatric disorders and on the patient’s characteristics in comparison to another three homologous periods of time (i.e., pre-lockdown, post-lockdown, and the time period in 2022, corresponding to the 2020 lockdown).

## 2. Materials and Methods

### 2.1. Study Design

We conducted an observational and retrospective analysis of inpatient psychiatric hospitalizations at the “Prof. Dr. Alexandru Obregia” Clinical Hospital of Psychiatry in Bucharest (Romania). The “Prof. Dr. Alexandru Obregia” Clinical Hospital of Psychiatry is a university teaching hospital and the largest psychiatric service in Romania. In addition to outpatient and day hospital services, the hospital provides inpatient emergency psychiatric care and covers a catchment area encompassing Bucharest, Romania’s capital city (with a population of almost 2 million inhabitants), and other neighboring counties. Moreover, the hospital is a tertiary center to which cases are referred from all over the country. The psychiatric emergency department is open 24 h on all days of the week and offers psychiatric care, including inpatient emergency care, for all individuals, regardless of their health insurance status.

We included only adult patients (aged over 18 years at the time of admission) admitted during specific time periods and with a primary diagnosis upon discharge falling under one of the following categories of disease and coded according to the 10th revision of the International Classification of Diseases (ICD-10): depressive disorder (F32, F33), bipolar disorder (F31), schizophrenia and schizoaffective disorder (F20, F25), acute psychotic disorders (F23), and delusional disorder (F22). The following data were collected from the hospital’s electronic database: age, gender, hospital length of stay (LOS), type of admission (voluntary or involuntary), and the primary diagnosis upon discharge. Records with incomplete data were excluded. All admissions were included (first hospitalization and readmission) in order to best reflect the utilization of the psychiatric inpatient services.

As mentioned before, the COVID-19 lockdown was implemented in Romania starting on 16 March 2020. The lockdown measures have been progressively curtailed since 15 May 2020 and were never reinstated. Only sanitary and physical distancing regulations have been maintained. Starting on 8 March 2022, the state of emergency ceased, and all restrictions or regulations related to the COVID-19 pandemic were cancelled. On this basis, four time periods were selected for this study:

- Pre-lockdown: from 16 January 2020 to 15 March 2020;
- Lockdown: from 16 March 2020 to 15 May 2020;
- Post-lockdown 2020: from 16 May to 15 July 2020;
- Post-lockdown 2022 (time period in 2022, corresponding to the 2020 lockdown): from 16 March 2022 to 15 May 2022.

The study received approval from the Institutional Research Ethics Committee of “Prof. Dr. Alexandru Obregia” Clinical Hospital of Psychiatry (approval no. 94/07.06.2022) and was carried out in accordance with the Declaration of Helsinki.

### 2.2. Statistical Analysis

In this study, categorical, ordinal and continuous variables were introduced. Categorical data were expressed as the absolute frequency (number) and relative frequency (percentage). Continuous variables were expressed as the median and interquartile range [Q1, Q3]. All data were tested for their normality of distribution using the Kolmogorov–Smirnov test. After cross-tabulation, the Chi-square test was used to study the correlations between the categorial variables. For the continuous variables, the Mann–Whitney U test (for two independent continuous variables) and Kruskal–Wallis test (for three or more continuous variables) with Dunn’s post hoc analysis were performed. The trend analysis was carried out using the Jonckheere–Terpstra test for ordered differences. For all tests, a *p* value < 0.05 was considered to be statistically significant (two-tailed). The statistical analysis was performed using the IBM Statistical Package for Social Sciences (SPSS) version 20.0 software for Windows (IBM, Armonk, NY, USA). 

## 3. Results

A total of 6604 patients who met the inclusion criteria were admitted to the “Prof. Dr. Alexandru Obregia” Clinical Hospital of Psychiatry during the studied timeframes. The admissions were divided into four groups according to the selected periods of time, as reported in Table 1: pre-lockdown—2787 patients, lockdown—840 patients, post-lockdown, 2020—1611 patients, post-lockdown, 2022—1366 patients. 

During the lockdown period, more than half of the patients with psychotic disorders were males (52.7%). Moreover, in the lockdown, post-lockdown 2020, and post-lockdown 2022 groups, most of the patients were involuntarily admitted (67.1%, 55.8%, and 54.5%, respectively). On the other hand, in the pre-lockdown group, 63.9% of the patients with psychotic disorders were voluntarily admitted. In the lockdown group, the patients with psychotic disorders had a median age of 43 [35, 53] years and a median LOS of 13 [8, 19.75] days. The median ages of the patients from the pre-lockdown and post-lockdown 2020 group were 46 [36, 56] years and 44 [36, 54] years, respectively. Moreover, in all four groups, most of the patients with psychotic disorders had a diagnosis in the category of schizophrenia and schizoaffective disorder (Table 1). Regarding affective disorders in the lockdown group, 53.3% of the patients were males and 39.4% were involuntarily admitted. On the contrary, during the pre-lockdown, post-lockdown 2020 and post-lockdown 2022 periods, 11.9%, 23.8%, and 20.1% of patients, respectively, were hospitalized by involuntary means. In the lockdown group, patients had a median LOS of 8 [5, 14.75] days. Patients from the pre-lockdown and post-lockdown 2020 groups had LOSs of 9 [6, 13] days and of 10 [7, 16] days, respectively. Other socio-demographic, clinical, and administrative characteristics of the patients are presented in Table 1.

In the psychotic disorders group, during the lockdown period, there was a significant decrease in the number of admissions in comparison with the pre-lockdown and post-lockdown 2020 groups (−53.31%, *p* < 0.001 and −23.55%, *p* < 0.001, respectively) (Table 2). Moreover, when compared with the pre-lockdown period and post-lockdown 2020 period, the number of admissions for a psychotic disorder significantly declined by 47.73% and 31.63%, respectively, in the post-lockdown 2022 period (*p* < 0.001 and *p* < 0.01, respectively). The trend analysis revealed that the admissions for psychotic disorders exhibited a descending trend (*p* < 0.001) (Table 1). When analyzed with respect to the diagnosis, the cases of hospitalized patients with schizophrenia during the lockdown period were fewer than those during the pre-lockdown or post-lockdown 2020 periods (−55.10%, *p* < 0.001 and −38.68%, *p* < 0.001, respectively). Regarding the cases of acute psychotic disorder, the number of patients declined by 49.06% during the lockdown period in comparison with the pre-lockdown (*p* < 0.001) period, but the number of patients increased by 68.38% during the post-lockdown 2020 period (*p* < 0.001). During the lockdown and post-lockdown period of 2022, the age of the hospitalized patients with psychotic disorders decreased in comparison with the pre-lockdown group (*p* < 0.05 and *p* < 0.01, respectively) (Table 2). No differences were observed between all periods of time in regard to the LOS of the patients with psychotic disorders (*p* > 0.05). During lockdown, it can be observed that the number of involuntary admissions declined by 13.11% in comparison with the pre-lockdown period (*p* < 0.001), but no statistically significant differences were observed in comparison with the post-lockdown 2020 and post-lockdown 2022 periods (*p* > 0.05 and *p* > 0.05, respectively). Moreover, during lockdown, there was a reduced number of hospitalized males compared to the pre-lockdown and post-lockdown 2020 periods (*p* < 0.001 and *p* < 0.01, respectively). Similar results were observed for females (*p* < 0.001 and *p* < 0.001, respectively) (Table 2).

As for the affective disorder group, the number of patients decreased in the lockdown subgroup when compared to the pre-lockdown group, post-lockdown 2020 group, and post-lockdown 2022 group (*p* < 0.001, *p* < 0.001, and *p* < 0.001, respectively) (Table 2). However, between the post-lockdown 2020 and post-lockdown 2022 periods, there was no statistical difference in terms of the number of hospitalized affective disorder cases (*p* > 0.05). The trend analysis revealed a descending trend for hospitalizations for affective disorders (*p* < 0.001) (Table 1). The LOS of the patients admitted during the post-lockdown 2020 period was significantly higher than that of the patients admitted in the lockdown or pre-lockdown periods (*p* < 0.001 and *p* < 0.001, respectively). Moreover, the median age of the patients admitted during lockdown and the post-lockdown period of 2020 for an affective disorder decreased in comparison with the pre-lockdown period (*p* < 0.001 and *p* < 0.001, respectively). In terms of the type of admission, the number of involuntary admissions significantly decreased only during lockdown, as compared to the pre-lockdown period (*p* < 0.01). It can be noticed that the number of admissions for bipolar disorder during lockdown was lower than that during the pre-lockdown or post-lockdown 2020 periods (*p* < 0.001 and *p* < 0.001, respectively). Moreover, between the pre-lockdown period and post-lockdown 2020 period, there was no statistical difference in terms of the number of bipolar patients (*p* > 0.05), but there was a statistical difference in terms of the depressive disorder cases (*p* < 0.001) (Table 2).

## 4. Discussion

The present research demonstrated that the inpatient admissions for all the studied diagnoses (i.e., schizophrenia and schizoaffective disorder, acute psychotic disorders, depressive disorder, and bipolar disorder), with the exception of delusional disorder, exhibited a significant decline during the imposition of lockdown measures. However, after the lockdown measures started to be gradually removed, there was a compensatory increase in the number of hospitalizations for psychotic or affective disorders, but it never exceeded the pre-pandemic levels. Moreover, our study reported that, during lockdown, inpatients with affective disorders or psychotic disorders were younger than those admitted during the pre-pandemic or immediate post-lockdown periods. Interestingly, our data revealed that the number of involuntary admissions for psychotic disorders in the lockdown period did not differ from that of the pre-pandemic period but, instead, there were many more compulsory hospitalizations after the lockdown was lifted. To our knowledge, this is the first study in Romania that analyzed the changes in these clinical and administrative parameters between different COVID-19 pandemic-related timeframes.

Until now, several studies have shown that the COVID-19 lockdown period was characterized by a significant reduction in the overall number of psychiatric inpatient admissions [13,15,16,20,21,22,23,24,25]. Our research is in line with these previous results. On the other hand, other studies identified no significant differences in the number of hospitalized patients [14,19,26,27]. Moreover, we found that the overall reduction in admissions affected all diagnoses, with the exception of delusional disorder. Lockdown regulations, especially travel restrictions, are probably the most important explanations for this finding, even though medical emergencies were obviously exempt from the restrictions. The fear of contagion is a reason of great importance that may explain patients’ reluctance to accept hospitalization. Indeed, studies that sought to identify the reasons for patients’ avoidance of hospital care identified the fear of contracting COVID-19 and the belief that there is a higher risk of getting infected in a health institution as possible answers to this question [28,29]. Moreover, a recent review pointed out the fact that individuals with mental health problems are at an increased risk of experiencing COVID-19-related fear [30]. Therefore, we can argue that all of these aspects may have hindered individuals with mental health problems from seeking treatment during lockdown, especially in the form of hospitalization. An important role in the emergence of such factors has been played by the mass media. As Clerici et al. (2020) and Boldrini et al. (2021) already argued, the mass media heavily outlined the higher risk of contracting COVID-19 in hospital settings, thus changing attitudes and behaviors towards inpatient admission [16,20]. These reasons could presumably also explain the reduced length of stay for affective disorders. Albeit anecdotal, one possible reason for this outcome may be the tendency of doctors to discharge patients earlier than usual due to fears of hospital-acquired COVID-19 infection. Another justification for this marked reduction in hospitalizations is the Romanian government’s decision to restrict psychiatric admission criteria to only those cases that could be considered as emergencies in order to prevent contagion in health facilities. This decision was suspended during the immediate post-lockdown period and also in the post-pandemic period when, similarly, a decrease in the number of hospitalizations compared to the pre-pandemic period was observed. Therefore, the drop in psychiatric admissions during the lockdown period can be mostly attributed to the fear of contracting the COVID-19 virus. Moreover, the descending trend observed for both psychotic and affective disorders contributes to evidence supporting the possible long-term effects of hospital avoidance attitudes and fear of infection. Above all, country-specific administrative regulations of the healthcare sector are an important factor to be considered. Future research is needed in order to shed further light on these matters and propose possible solutions.

Regarding the type of admission (i.e., voluntary or involuntary), we found that voluntary hospitalizations significantly decreased during the COVID-19 lockdown in both diagnostic categories (i.e., psychotic disorders and affective disorders). Previous research, which identified an overall reduction in voluntary psychiatric admissions, is consistent with our results [12,13,20,21], although data at variance with ours has been reported in the literature [15,16,31]. In addition, the percentage of involuntary admissions for psychotic disorders increased from 11.9% in the pre-pandemic period to 39.4% during lockdown, which is similar to the results reported in another study [24]. Nevertheless, the decline in psychiatric admissions during lockdown can mainly be attributed to the decline of voluntary admissions. Besides the fear of a higher chance of contracting the virus in a hospital setting and the policy of cancelling nonurgent admissions, which are voluntary by default, visiting restrictions could have been another cause of these changes. Interestingly, Romanian individuals seem to have developed an increased sense of loneliness during the national lockdown [32]. Therefore, such restrictions not only affected patients’ satisfaction with the quality of care [33] but, considering this increased sense of loneliness, we suggest that they may have also prevented patients from seeking inpatient services for fear of losing connection with family and loved ones. Another explanation for the reduced number of admissions during lockdown compared with the pre-lockdown period, and also for the descending trend in the psychiatric hospitalizations for all diagnostic groups (affective and psychotic), is the reduction in the number of available beds. Similar to hospitals from other countries [34,35], this measure was employed in our hospital with the purpose of increasing the distance between patients and, therefore, maintaining epidemiological safety. Additionally, a German national survey study aimed at exploring the challenges involved in inpatient psychiatric care during the COVID-19 pandemic showed that the two most important reasons for the reduced bed occupancy rate were the efforts to enable isolation and maintain physical distancing and to ensure protection of the patients and staff in general [36]. 

Our report also revealed a post-lockdown increase in the number of total admissions for all the diagnostic categories. Moreover, the involuntary admissions for psychotic disorders increased, and that for affective disorders returned to pre-pandemic levels. A similar study conducted at a university hospital in Geneva, Switzerland, reported results that are in agreement with ours [37]. Numerous studies have provided evidence of the profound negative impacts of the COVID-19 pandemic and subsequent lockdown on the general population. The COVID-19 lockdown seems to have elicited an increased burden of psychological and psychiatric problems, which mainly include higher levels of stress, anxiety, and perceived loneliness, depressive symptoms, sleep disturbances, and the disruption of social connectedness [7,8,38]. These problems have persisted or even increased in different countries, despite lockdown restrictions being lifted [39,40]. Notably, a recent meta-analysis identified a significant increase in the prevalence of major depressive disorder and anxiety disorders between the pre-pandemic and pandemic periods [7]. Moreover, the diagnosis of a psychotic disorder is a risk factor for involuntary hospitalization [41]. In addition, lower income, financial problems, and unemployment are issues that arose immediately after the outbreak started and are still present in post-pandemic society [42]. Not to mention that all of these social and psychological consequences are already established risk factors for a wide range of psychiatric disorders [43,44]. Against this background, we can assume that the increase in inpatient admissions during the post-lockdown period might mirror these consequences, since the utilization of inpatient psychiatric care acts as an indicator of the mental health of the general public [45]. 

Even though, in our study, there were differences in terms of the numbers of hospitalizations between the lockdown and post-lockdown periods in favor of the latter, there was a descending trend compared to the pre-pandemic period. One explanation for this trend could be the fact that there are different periods in the year and this seasonal effect could have biased our data interpretation. However, the subject of seasonality is still under debate and no clear conclusions have been drawn thus far [46,47]. Another hypothesis is related to the possible paradoxical effect of the reorganization of the mental healthcare services carried out during lockdown. Before the COVID-19 pandemic, psychiatric hospitals were one of the most important providers of mental health services in Romania. Many factors could explain this state of affairs, but the most important are the human resource of psychiatrists, which is below that of other European countries and unequally distributed [48], the traditional attitude of the Romanian people to seeking treatment in the form of hospitalization, and the hospital-centric organization of the system, despite a functional network of community mental health centers. Since hospital-based mental healthcare was largely reduced during the whole pandemic period, we observed an enhanced activity and outreach capacity of outpatient facilities after the lockdown ended, as well as an increased involvement of primary care in the provision of psychiatric care. Moreover, a possible change in people’s attitudes toward hospital-based care might have had an important impact on inpatient admissions. Further research is needed to test these hypotheses and to unveil the maze of interactions behind these changes. 

Younger people are a special category who are more vulnerable to stress and who were at an increased risk of mental health problems during the COVID-19 pandemic and lockdown, as outlined by different research reports [7,49,50,51]. Nejati et al. (2021) observed an increase in admissions among the 35–44 age group [27]. Additionally, decreased age was found to predict inpatient admissions during lockdown [25]. Our results support these findings, as we observed a decrease in the age of the hospitalized patients during lockdown. Coupled with the aforementioned reasons, other possible explanations include the doctors’ reluctance to admit older patients due to concerns of the safety of hospital environments and the increased COVID-19 mortality among this particular population [52]. 

The LOS can be viewed as a measure of illness severity. Against normative expectations, our results showed that the LOS of patients with psychotic disorders did not increase in the post-lockdown period in comparison with the confinement period. Moreover, there was again no difference in this parameter when compared to the pre-pandemic period. Taken together, these data reveal that the number of days of hospitalization was more related to the course of the psychotic illnesses, and the psychological stressors stemming from the COVID-19 lockdown exerted a minimal influence on this parameter in the short term. In addition, these results highlight the successful efforts of primary care and outpatient services to ensure the continuity of care of patients with chronic mental disorders. However, based on the knowledge amassed from past catastrophic events, psychological distress can determine psychiatric consequences (i.e., the onset or relapse of psychotic disorders) in the long term [53]. 

This study has several limitations that should be acknowledged. First of all, due to the retrospective and cross-sectional nature of the research, we could not directly assess the patients in order to establish the diagnosis using a structured clinical interview. Moreover, due to the retrospective nature of the data collection in this study, we could not analyze certain variables, such as symptom severity, suicidality, history of mental health problems, COVID-19 infection, ongoing treatments, etc. Secondly, the diagnoses were established by different psychiatrists. It is important to mention that the psychiatrists from our hospital are well familiarized with the ICD-10 criteria and encoding system, since it has been implemented for many years in our hospital. Moreover, we analyzed the diagnosis at discharge, which, unlike an emergency/admission diagnosis, is made after a thorough follow-up. Thirdly, we did not report the patients with comorbid psychiatric diagnoses. Drug addiction, in particular, together with other comorbid psychiatric disorders, is an important factor in the frequent use of inpatient psychiatric services and contributes to the “revolving door” phenomenon [54]. Finally, even though the studied population was large, this research was conducted in a single psychiatric service; thus, this prevents us from generalizing our results to the entire Romanian mental healthcare system.

## 5. Conclusions

In conclusion, the present study demonstrates the important impacts of the COVID-19 pandemic on inpatient admissions for severe mental illnesses and adds a piece to the puzzle of the current global mental health question. Taken together, our findings demonstrate a decrease in the utilization of hospital services for psychotic and affective disorders during the COVID-19 lockdown and also indicate a negative trend in admissions throughout the COVID-19 pandemic. Moreover, the younger age of the patients hospitalized during lockdown might reflect a certain vulnerability of this group, which calls for further analysis. Future prospective replication studies are required to confirm our results and enrich the currently scarce knowledge regarding the immediate and long-term impacts of the COVID-19 pandemic on the mental health of the Romanian population. Moreover, these instances call for reflection on how we can improve the mental health system, mitigate disparities in care provision, and address the impacts of the COVID-19 pandemic. The implementation of preventive mental health strategies (i.e., coherent screening programs and easily available psychological educational resources), improvement of access to services (i.e., the enhancement of community, crisis, and home-treatment teams and the further development of tele-medicine services), and the shift towards an integrated primary care model represent reliable solutions. Given that, to the best of our knowledge, this is the first research of its kind conducted in Romania, our study could serve as a starting point for health professionals and policy makers to form an immediate action plan.

## Figures and Tables

**Table 1:** Socio-demographic, clinical, and administrative characteristics of psychiatric admissions during the COVID-19 lockdown period in comparison with the pre-lockdown period, post-lockdown period, and the lockdown corresponding period in 2022.

	Pre-Lockdown (*n* = 2787)	Lockdown (*n* = 840)	Post-Lockdown 2020 (*n* = 1611)	Post-Lockdown 2022 (*n* = 1366)	p *, #
Psychotic Disorders					
Number of admissions (*n* = 2619)	951	444	727	497	* < 0.001 # < 0.001
Gender, *n* (%)					
Females	548 (57.6)	210 (47.3)	376 (51.7)	270 (54.3)	<0.001
Males	403 (42.4)	234 (52.7)	351 (48.3)	227 (45.7)	<0.001
Age (years), median [Q1, Q3]	46 [36, 56]	43 [35, 53]	44 [36, 54]	44 [35, 52]	=0.001
LOS (days), median [Q1, Q3]	12 [7, 18]	13 [8, 19.75]	13 [8, 20]	13 [8, 17]	>0.05
Type of admission, N (%)					
Voluntary	608 (63.9)	146 (32.9)	321 (44.2)	226 (45.5)	<0.001
Involuntary	343 (36.1)	298 (67.1)	406 (55.8)	271 (54.5)	<0.001
Diagnosis, N (%)					
Schizophrenia and schizoaffective disorder	646 (67.9)	290 (65.3)	473 (65.1)	327 (65.8)	<0.001
Acute psychotic disorders	267 (28.1)	136 (30.6)	229 (31.5)	154 (31)	<0.001
Delusional disorder	38 (4)	18 (4.1)	25 (3.4)	16 (3.2)	>0.05
Affective Disorders					
Number of admitted patients (*n* = 3985)	1836	396	884	869	* < 0.001 # < 0.001
Gender, *n* (%)					
Females	947 (51.6)	185 (46.7)	508 (57.5)	479 (55.1)	<0.001
Males	889 (48.4)	211 (53.3)	376 (42.5)	390 (44.9)	<0.001
Age (years), median [Q1, Q3]	55 [44, 63]	49 [41, 59]	54 [43, 63]	52 [41, 62]	<0.001
LOS (days), median [Q1, Q3]	9 [6, 13]	8.5 [5, 14.75]	10 [7, 16]	9 [6, 14]	<0.001
Type of admission, *n* (%)					
Voluntary	1617 (88.1)	240 (60.6)	674 (76.2)	694 (79.9)	<0.001
Involuntary	219 (11.9)	156 (39.4)	210 (23.8)	175 (20.1)	<0.01
Diagnosis, *n* (%)					
Depressive disorder	1563 (85.1)	291 (73.5)	691 (78.2)	718 (82.6)	<0.001
Bipolar disorder	273 (14.9)	105 (26.5)	193 (21.8)	151 (17.4)	<0.001

* Kruskal–Wallis test; # Jonckheere–Terpstra test; LOS, length of stay; *n*, number of patients; Q, quartile.

**Table 2:** Comparison of the socio-demographic, clinical, and administrative variables of the psychotic disorder and affective disorder groups between the respective time periods.

	Lockdown vs. Pre-Lockdown	Lockdown vs. Post-Lockdown 2020	Lockdown vs. Post-Lockdown 2022	Pre-Lockdown vs. Post-Lockdown 2020	Pre-Lockdown vs. Post-Lockdown 2022	Post-Lockdown 2020 vs. Post-Lockdown 2022
Psychotic disorders						
Number of patients admitted	<0.001 (−53.31%)	<0.001 (+63.73%)	>0.05	>0.05	<0.001 (−47.73%)	<0.01 (−31.63%)
Gender						
Females	<0.001	<0.001	>0.05	<0.05	<0.001	<0.05
Males	<0.001	<0.01	>0.05	>0.05	<0.001	=0.001
Age	<0.05	>0.05	>0.05	>0.05	<0.01	>0.05
LOS	-	-	-	-	-	-
Type of admission						
Voluntary	<0.001 (−75.98%)	<0.001 (+119.86%)	>0.05	=0.001 (−47.20%)	<0.001 (−62.82%)	>0.05
Involuntary	<0.001 (−13.11%)	>0.05	>0.05	>0.05	>0.05	<0.001 (−33.25%)
Diagnosis						
Schizophrenia and schizoaffective disorder	<0.001 (−55.10%)	<0.001 (+63.10%)	>0.05	<0.05	<0.001 (−49.38%)	<0.05 (−30.86%)
Acute psychotic disorder	<0.001 (−49.06%)	<0.001 (+68.38%)	>0.05	>0.05	<0.001 (−42.32%)	<0.01 (−32.75%)
Delusional disorder	-	-	-	-	-	-
Affective Disorders						
Number of patients admitted	<0.001 (−78.43%)	<0.001 (+123.23%)	<0.001 (+119.44%)	=0.001 (−51.85%)	<0.001 (−52.66%)	>0.05
Gender						
Females	<0.001	<0.001	<0.001	<0.05	<0.01	>0.05
Males	<0.001	<0.001	<0.001	<0.001	<0.001	>0.05
Age	<0.001	<0.001	>0.05	>0.05	<0.001	>0.05
LOS	>0.05	<0.001	>0.05	<0.001	<0.05	<0.01
Type of admission						
Voluntary	<0.001 (−85.15%)	<0.001 (+180.83%)	<0.001 (+189.16%)	<0.001 (−58.31%)	<0.001 (−57.08%)	>0.05
Involuntary	<0.01 (−28.76%)	>0.05	>0.05	>0.05	>0.05	>0.05
Diagnosis						
Depressive disorder	<0.001 (−81.38%)	<0.001 (+137.45%)	<0.001 (+146.73%)	<0.001 (−55.79%)	=0.001 (−54.06%)	>0.05
Bipolar disorder	<0.001 (−61.53%)	<0.001 (+83.80%)	>0.05	>0.05	=0.001 (−44.68%)	>0.05

Kruskal–Wallis test with Dunn’s post hoc analysis; LOS, length of stay.

## Data Availability

The data presented in this study are available on reasonable request from the corresponding authors. The data are not publicly available due to ethical and institutional reasons.
